# Timeliness and Equity: An Analysis of Measles Herd Immunity in a Regional Area of Australia

**DOI:** 10.3390/vaccines14010001

**Published:** 2025-12-19

**Authors:** Megan Whitley, Katrina Clark, Michelle Butler, Peter Murray, Hannah Briggs, Sharon Saxby, David N. Durrheim

**Affiliations:** Health Protection Unit, Hunter New England Local Health District, NSW Health, Wallsend NSW 2287, Australia

**Keywords:** measles, immunisation, timeliness, equity, Aboriginal, coverage, elimination, herd immunity

## Abstract

Background: Global declines in immunisation rates and a resurgence in measles pose a threat, even in countries like Australia that have achieved elimination status. National coverage in Australia is measured at static timepoints, so it is unclear at what age children received their vaccines. This may permit the emergence of immunity gaps, leaving children susceptible to measles between those reporting timepoints. Methods: A cross-sectional retrospective analysis was conducted using routinely collected data from the Australian Immunisation Register for children residing in Hunter New England Local Health District (HNELHD), New South Wales, born from 1 January 2015 to 1 June 2019 as a quality improvement initiative. Coverage, age at immunisation, and on-time immunisation were described by demographic, local geographic and age variables. Reverse survival analysis was conducted to determine the timing of achieving 95% MCV2 coverage. To ensure the cultural integrity of the research, an Aboriginal researcher co-led the design, analysis and interpretation of results. Results: The analysis included 53,390 children. Measles coverage exceeded the national and international target of 95% MCV2 coverage, with coverage in Aboriginal children surpassing national rates for all children. Pockets of low coverage were identified in several smaller geographic areas and subpopulations. Median age of MCV1 receipt was 375 days (IQR: 369–390 days), and MCV2 was 560 days (IQR: 551–583 days). More recent birth cohorts had earlier immunisation. On-time immunisation rates were high, and most children receiving measles immunisation late were still immunised within six months of the schedule date. The 95% MCV2 coverage threshold was achieved at 1582 days of age. Conclusions: Robust measles immunisation coverage and timeliness were found in HNELHD, Australia. Timeliness data analysis is a useful adjunct to static coverage data in understanding immunisation protection. Improving immunisation data availability, accessibility, and timeliness offers potential to better inform targeted public health activity.

## 1. Introduction

Measles causes significant morbidity and mortality globally and is targeted for elimination in all World Health Organization (WHO) regions [1,2,3]. People of all ages can be affected; however, measles is an important childhood disease. Young children are particularly vulnerable to respiratory, gastrointestinal and neurological complications. Most deaths from measles occur in children under five who are under- or unvaccinated [2,4,5]. Infection with measles leaves children at longer-term risk of other infections, through transient but profound immunosuppression and impairment of humoral immune memory [2]. A safe and effective live attenuated vaccine has been available in Australia since 1968. The vaccine confers lifelong immunity in almost all recipients after two doses [6,7]. As measles is highly infectious, a 95% second dose measles (MCV2) coverage threshold must be reached to achieve and sustain measles elimination, and it is an indicator of herd immunity [2,8,9,10].

The current global resurgence of measles poses a threat to health and elimination targets [10]. All WHO regions have reported an increase in case numbers since 2024, with large, disruptive outbreaks occurring in several countries [11]. Global first-dose measles-containing vaccine (MCV1) coverage dropped from 86% in 2019 to 84% in 2024. An estimated 20.6 million infants did not receive their first routine immunisation dose in 2024 [12,13]. Disruptions to regional elimination efforts were preceded by interruptions to routine and catch-up immunisation programmes during the COVID-19 pandemic, which increased vaccine hesitancy, health system resource constraints, and changing geopolitical landscapes (including funding cuts to global public health programmes) [5,10,11].

Australia achieved elimination of endemic measles in 2014 but continues to import the virus through travellers [10]. By mid-year, the 2025 case numbers already surpassed the total 2024 numbers, with multiple importations particularly from Southeast Asian countries experiencing outbreaks [3,14,15].

In Australia, measles immunisation is funded by the National Immunisation Programme, providing free MCV at 12 and 18 months of age. Vaccine effectiveness is estimated at 96.7% after one dose and 99.7% after two doses [7,8]. The schedule has been in place since 2013 [6]. The programme is supported by a number of policies and catch-up programmes. However, opportunities exist to improve coverage [16,17].

Maintaining vaccination coverage is a priority at national, state, and local levels [18,19]. National coverage reports are made publicly available annually in Australia, providing data at a sub-national level [17]. While coverage and on-time immunisation are included, the actual age at which children are vaccinated is not given. Reporting measures coverage at fixed timepoints to determine immunisation status. However, the timepoints (12, 24, and 60 months) leave potential gaps, during which children may be susceptible. In addition, children who are immunised beyond these timepoints may not be captured until the next timepoint, providing an incomplete picture of true coverage rates [17]. State-based performance measures assess full immunisation at the 12–15 months and the 60–63-month timepoints [20]. In 2024, 94.0% of Australian children received MCV1 by 24 months, 91.4% received MCV2 by 24 months, and 96.1% received MCV2 by 60 months [17]. Aboriginal children had higher MCV1 coverage by 24 months (94.7%) and MCV2 by 60 months (97.9%), but lower MCV2 coverage at 24 months (89.5%), raising equity concerns. On-time immunisation (those given within 30 days of the recommended date) rates are declining, with only 65.4% of all children and 55.3% of Aboriginal children having received MCV1 on time nationally in 2024 [17,21].

This study is based in the Hunter New England Local Health District (HNELHD) in northeastern New South Wales, Australia. HNELHD is located on the traditional Aboriginal lands of Kamilaroi, Gomilaroi, Geawegal, Bahtabah, Thungutti, Awabakal, Aniawan, Biripi, Worimi, Nganyaywana, Wonnarua, Banbai, Ngoorabul, Bundjalung, Yallaroi and Darkinung people [22]. We investigated potential immunity gaps among children aged five years and under due to delayed immunisation or coverage gaps. This work was undertaken as a quality improvement initiative. HNELHD is a large geographic region encompassing metropolitan, regional and rural areas, with a population of over 970,000 people. Approximately seven percent of the population identifies as Aboriginal [22]. Given the diverse population of HNELHD, we were concerned that immunity gaps may adversely affect subpopulations within the region, including children who were due for their MCV1 and MCV2 in the early COVID-19 pandemic period.

## 2. Materials and Methods

A cross-sectional retrospective analysis was conducted using routinely collected Australian Immunisation Register (AIR) data for children resident in HNELHD. The analysis sought to determine local patterns of coverage and timeliness, and identify any immunity gaps as a quality improvement initiative. The AIR is a whole-of-life record of immunisations given to all Australian residents. There is a legal requirement for immunisation providers to record doses administered under the National Immunisation Programme [23]. Previous reviews of the AIR data have demonstrated high levels of completeness, including ~99% of children registered by 12 months of age [24,25,26].

### 2.1. Cultural Governance

This study ensured strong cultural governance by embedding co-leadership of two Aboriginal researchers who were involved in all stages of the research, providing cultural governance, and ensuring cultural safety and integrity were maintained. An independent Aboriginal Reference Group provided additional cultural guidance and support for the duration of the study. Strengths-based approaches were used during the planning and interpretation, and reporting of data.

### 2.2. Data Sources

AIR data routinely available to the HNELHD Public Health Unit for quality assurance was the source dataset. Data was extracted from the AIR based on date of birth and for MCV given or overdue since 1 February 2016. Data was downloaded on 12 June 2025, so any changes in the source data are not reflected in this analysis.

The variables included, and their definitions, are shown in Table 1.

### 2.3. Inclusion and Exclusion Criteria

Inclusion criteria were a current HNELHD postcode within the AIR and a date of birth between 1 January 2015 and 1 June 2019. These criteria were selected to best capture all children residing in the region and therefore contributing to population immunity. This age range permitted follow-up until the child was five years of age (the static reporting timepoint).

Exclusion criteria were children registered on the AIR as deceased or having permanently left Australia, as they could no longer contribute to population immunity.

Duplicate episodes, rejected or excluded doses, or doses clarified and otherwise recorded (AIR codes X, Y, Z, R) were also omitted.

### 2.4. Geographic and Socioeconomic Classifications

Postcodes recorded in the AIR were used to determine local government area (LGA) of residence, Socio-Economic Indexes for Areas (SEIFA), and Accessibility/Remoteness Index of Australia (ARIA). Postcodes and LGAs were compared with SEIFA and ARIA datasets. SEIFA was calculated using the Index of Relative Socio-Economic Advantage and Disadvantage. The results were classified into deciles, where the highest scoring decile indicated a relative lack of disadvantage and greater advantage, and a low score indicated a greater relative disadvantage and a lack of advantage [27]. There are no areas in HNELHD in the SEIFA 10 decile. ARIA areas were based on the Australian Statistical Geography Standard (ASGS) remoteness structure into classes of relative geographic remoteness [28]. In HNELHD, there are no areas classified as Remote or Very Remote Australia.

### 2.5. Types of Vaccine

Vaccines were grouped according to their antigen component as measles, mumps and rubella (MMR), and measles, mumps, rubella and varicella (MMRV), given that brands can be used interchangeably. Vaccines received overseas refer to any measles-containing vaccine that was received outside of Australia.

There were no changes to the Australian measles immunisation recommendations or schedule during the study period [6].

### 2.6. Data Cleaning and Management

Data was cleaned in R, and then housed in a secure REDCap project with de-identified data available for extraction by the research team.

### 2.7. Descriptive and Statistical Analysis

Statistical analysis was performed using open-source software R version 4.4.1 [29]. Microsoft Excel was also used to produce graphs.

Descriptive epidemiological analysis was performed using the variables described in Table 1, as well as vaccine type. Four categories were derived from the variable describing immunisations received after the scheduled date: received 31–60 days after the scheduled date, 61–120 days after the scheduled date, 121–183 days after the scheduled date, or 184 days or more after the scheduled date.

Age at immunisation was described in days for both doses. The age at the time of immunisation was positively skewed, so the median was taken as a measure of central tendency. The large dataset was not normally distributed, so non-parametric tests, including the Kruskal–Wallis test, were used. Where this was significant, a post hoc Dunn’s test was used to calculate and adjust the *p*-value. A *p*-value of <0.05 was considered significant.

A reverse survival analysis was conducted to determine when 95% coverage with two doses of MCV was achieved for the cohort (as an indicator of herd immunity).

## 3. Results

**Figure 1:**
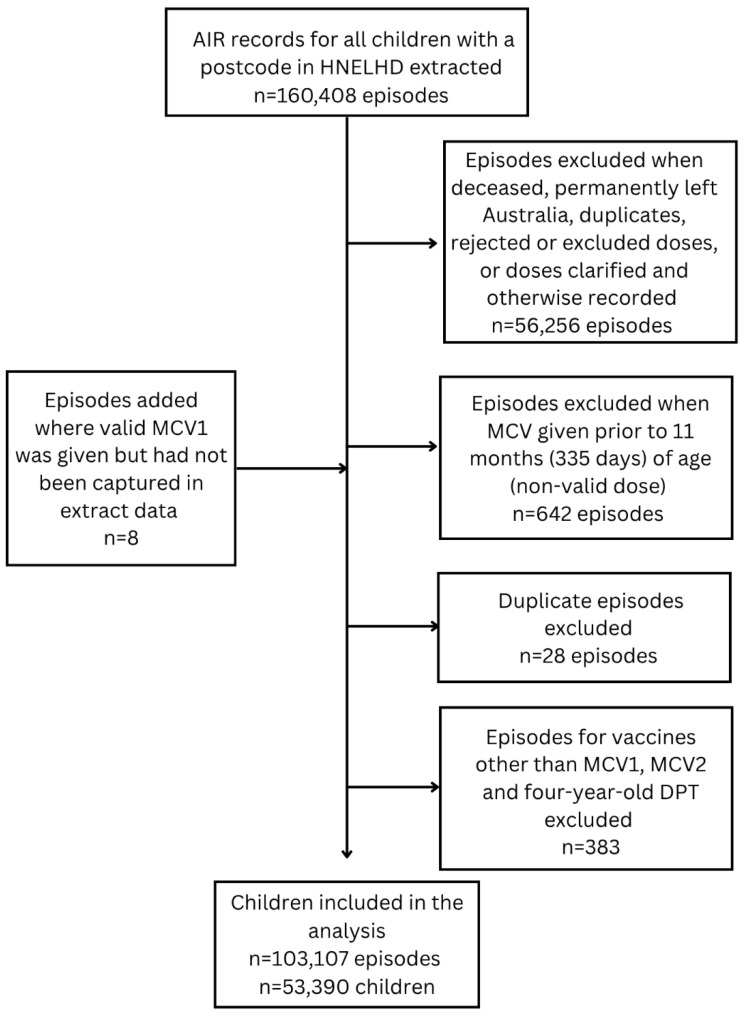
Flowchart of inclusion and exclusion for children in the study.

There were 53,390 children included in the analysis (Figure 1). Demographic details are included in Table 2. A total of 14.7% of children identified as Aboriginal (*n* = 7864), 84.6% were non-Indigenous (*n* = 45,189), and 0.6% had no Indigenous status recorded (*n* = 337). Completeness of recording of Indigenous status improved over time from 99.1% complete in the 2015 cohort to 99.6% in the 2019 cohort. There was a decline in birth cohort populations during the study period, and the proportion of children who identified as Aboriginal increased, from 13.3% in the 2015 birth cohort to 15.7% in the 2019 cohort. LGAs with the highest absolute number of children followed population density patterns, with most children residing in major cities. HNELHD has 63.7% of postcodes in the bottom 50% of the SEIFA index, indicating a relatively higher incidence of disadvantage.

### 3.1. Immunisation Coverage at the Time of Analysis

**Figure 2:**
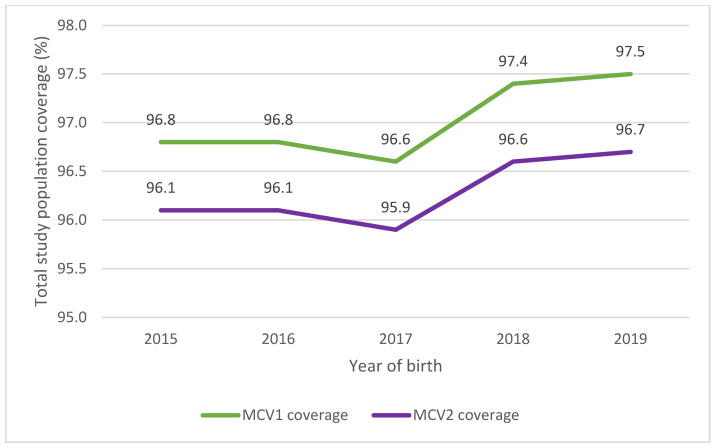
Coverage for MCV1 and MCV2 by year of birth for all children in HNELHD included in the study at the time of analysis.

Of the 53,390 children included, 1621 (3.0%) children did not have any valid measles immunisation recorded in the AIR; 51,769 (97.0%) children received at least one valid dose of MCV; and 51,361 (96.2%) received two valid MCVs. Coverage varied slightly by birth cohort, with the 2017 birth cohort having the lowest coverage and the 2019 cohort having the highest coverage (Figure 2).

Measles immunisation coverage was consistently higher across all birth cohorts for Aboriginal children compared with non-Indigenous children and children with no Indigenous status recorded. Aboriginal children had 98.9% coverage (*n* = 7781) for MCV1 and 98.5% (*n* = 7744) for MCV2, meaning 99.5% of Aboriginal children who received MCV1 received MCV2. Non-Indigenous children had 96.9% (*n* = 43,809) MCV1 and 96.2% (*n* = 43,472) MCV2 coverage, meaning 99.2% of those who had MCV1 received MCV2. Children whose Indigenous status was not recorded had low measles immunisation coverage recorded with 46.9% (*n* = 158) receiving no valid MCV, 53.1% (*n* = 179) receiving MCV1 and 43.0% (*n* = 145) receiving MCV2.

**Figure 3:**
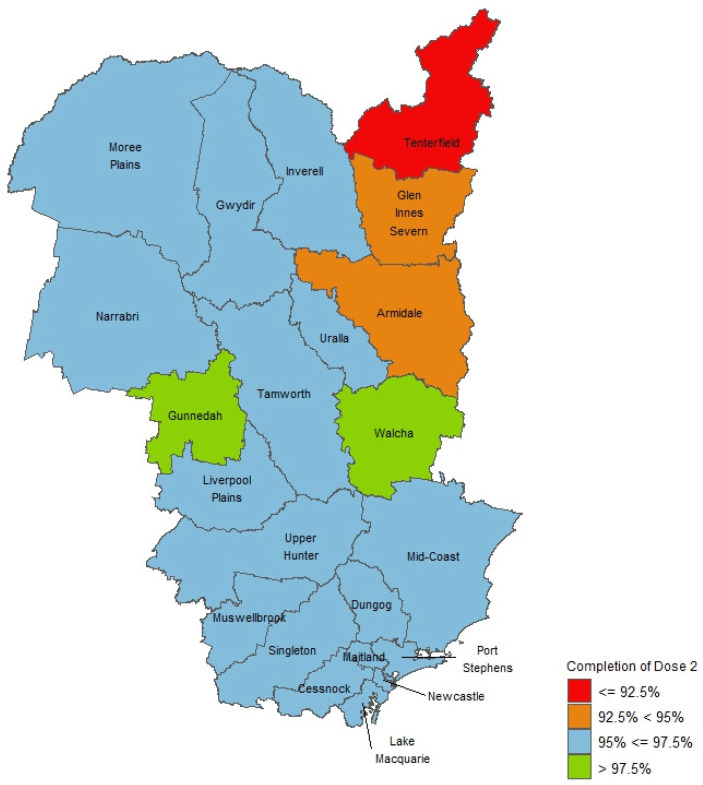
Map of MCV2 coverage for children in HNELHD by local government areas during the study period.

Measles immunisation coverage varied by LGA (Figure 3), with three regional LGAs in the northeastern part of HNELHD not achieving the 95% MCV2 coverage threshold during the study period (Tenterfield (91.6%), Armidale (93.6%), and Glen Innes Severn (94.3%). Inter-LGA variation by birth cohort was noted, which did not always follow HNELHD trends.

Inner regional areas had lower coverage (MCV1 96.7%, MCV2 95.9%) compared with major cities and outer regional areas (both MCV1 97.1%, MCV2 96.4%). An economic gradient was not observed for MCV coverage during the study period. Coverage was highest in SEIFA decile 3 for MCV1 (98.0%, *n* = 2535) and MCV2 (97.1%, *n* = 2511) and lowest in SEIFA decile 8 (MCV1 96.4% *n* = 2492; MCV2 95.5% *n* = 2467). All SEIFA deciles demonstrated MCV1 coverage above 95%.

### 3.2. Immunisation Timeliness

Timeliness of MCV1 and MCV2 was considered by age at immunisation in days, and whether the immunisation episode was on time or after the scheduled date.

#### 3.2.1. Age at Immunisation

The median age of receipt of MCV1 immunisation was 375 days (IQR: 369–390 days, range: 336–3679 days), with the scheduled on-time range being 365–395 days; however, doses are considered valid from 336 days. Median age of MCV2 was 560 days (IQR: 551–583 days, range: 389–3706 days), with scheduled on-time range being 548–578 days. Distributions for both MCV1 and MCV2 were positively skewed, with few immunisation episodes occurring late.

When considered by birth cohort, statistically significant differences were seen for age at immunisation between all birth cohorts for MCV1 and MCV2; however, the absolute difference in median age was marginal (two days for MCV1 and one day for MCV2) (Appendix A). Post hoc analysis demonstrated that the 2019 birth cohort had timelier immunisation than all other cohorts for MCV1 and timelier MCV2 than the 2016, 2017, and 2018 cohorts (*p* < 0.01, Appendix A). Analysis by Indigenous status showed small absolute differences in age at immunisation between Aboriginal and non-Indigenous children for MCV1 (median age 376 and 374 days, respectively) and MCV2 (median age 562 and 559 days, respectively) (*p* < 0.01, Appendix A). Children with no Indigenous status had a later median age of immunisation (401 days for MCV1 and 605 days for MCV2). Non-Indigenous children had timelier MCV1 and MCV2 than Aboriginal children, and those without Indigenous status recorded. Aboriginal children had timelier MCV1 and MCV2 compared with children without Indigenous status recorded (*p* < 0.01). Statistically significant difference was seen for age at immunisation for Indigenous status (Appendix A).

#### 3.2.2. On-Time or After-Scheduled-Date Immunisation

**Figure 4:**
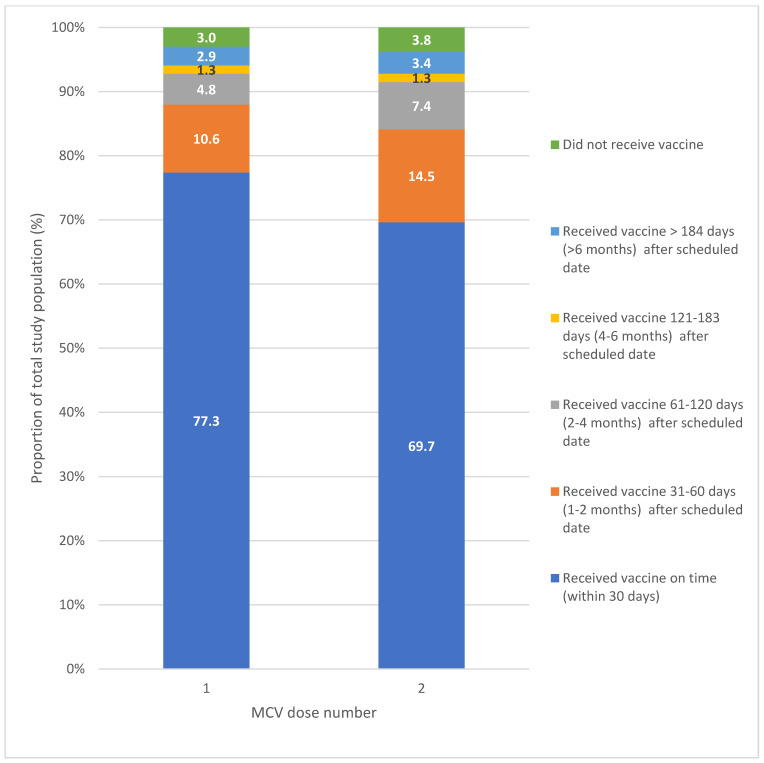
On-time and delayed immunisation for MCV1 and MCV2 for all children in the study.

For all children included in the study, 77.3% (*n* = 41,244) received MCV1, and 69.7% (*n* = 37,209) received MCV2 on time; 19.7% (*n* = 10,526) received MCV1 and 26.5% (*n* = 14,152) received MCV2 more than 30 days after the scheduled date; and 3.0% (*n* = 1620) did not receive MCV1 while 3.8% (*n* = 2029) did not receive MCV2 (Figure 4). For both doses, most children were immunised within six months after the scheduled date.

The proportion of on-time immunisation was highest for MCV1 in the 2019 birth cohort (81.0%, *n* = 4007) and for MCV2 in the 2015 birth cohort (70.3%, *n* = 8750). There was limited variation between birth cohorts for both MCV1 and MCV2; however, there was a declining proportion of doses given more than six months after the scheduled date.

**Figure 5:**
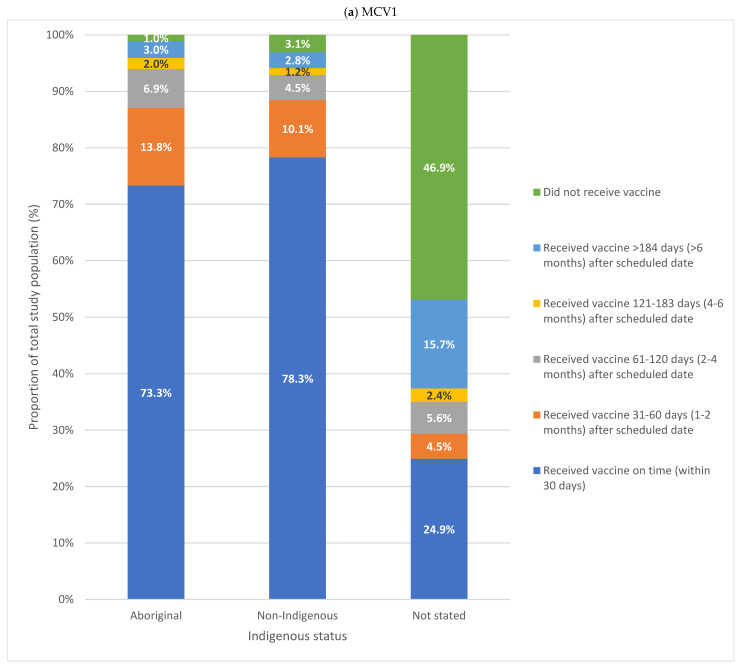

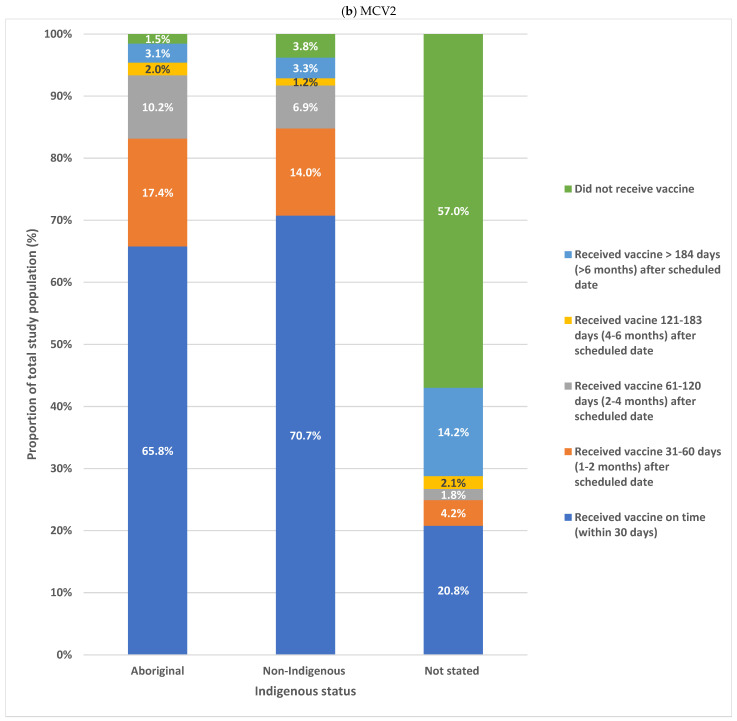
On-time and delayed immunisation for MCV1 (**a**) and MCV2 (**b**) by Indigenous status for all children in HNELHD during the study. Aboriginal children *n* = 7864; non-Indigenous children *n*= 45,189; children without Indigenous status recorded *n* = 337.

Greater variation in on-time immunisation for both MCV1 and MCV2 was noted when considering Indigenous status (Figure 5). Almost three-quarters of Aboriginal children and 78.3% of non-Indigenous children received MCV1 on time, with most of those immunised late receiving MCV1 within six months of the scheduled age. MCV2 was less timely, with 65.8% of Aboriginal children and 70.8% of non-Indigenous children immunised on time; however, most children received their MCV2 within six months of the scheduled age. Children without an Indigenous status recorded differed substantially, with low on-time immunisation and more delayed immunisation greater than six months.

There was variation between LGAs in on-time immunisation for MCV1 (ranging from 66.0% *n* = 165 in Tenterfield to 80.8% *n* = 1218 in Singleton LGAs) and MCV2 (ranging from 53.2% *n* = 133 in Tenterfield to 74.0% *n* = 228 in Uralla LGAs). Armidale LGA had a higher proportion of immunisations received more than six months after the scheduled date (8.3% *n* = 131 for MCV1, and 6.7% *n* = 106 for MCV2) compared with other LGAs.

Children living in major cities had a higher proportion of on-time immunisation (78.6% *n* = 24,147 for MCV1, and 71.2% *n* = 21,883 for MCV2) compared with children in inner regional areas (75.5% *n* = 13,676 for MCV1, and 67.6% *n* = 12,249 for MCV2) and outer regional areas (75.4% *n* = 3420 for MCV1, and 67.9% *n* = 3077 for MCV2).

Although a trend for a higher proportion of on-time immunisation for MCV1 and MCV2 in higher SEIFA deciles was noted, this was not statistically significant.

### 3.3. 95% MCV2 Coverage

**Figure 6:**
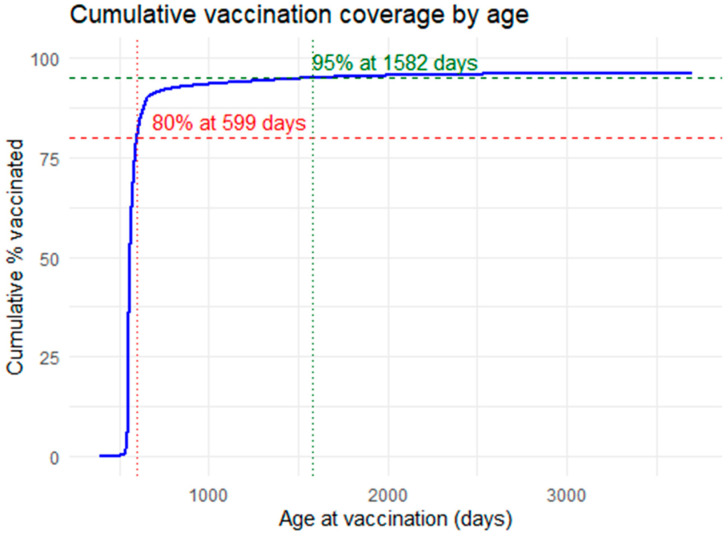
Time of 95% MCV2 immunisation coverage for HNELHD children in the study with 80% MCV2 coverage (red dotted line) and 95% MCV2 coverage (green dotted line) indicated.

During the study period, the 95% MCV2 coverage threshold was achieved for the entire cohort at 1582 days old, or four years and four months old (Figure 6). Eighty percent coverage was achieved at 599 days, or one year and seven months of age.

### 3.4. Vaccine Type

The majority of MCV1 was provided as MMR (97.0%, *n* = 50,247) and MCV2 as MMRV (97.2%, *n* = 49,935). MMRV was given to children aged under four years of age as MCV1 in 0.7% (*n* = 341) of doses. More children received MCV1 overseas (2.1%, *n* = 1064) than MCV2 (0.9%, *n* = 472). Children with no Indigenous status recorded had a higher proportion of vaccines received overseas (MCV1 29.1%, MCV2 15.0%).

## 4. Discussion

Maintaining high and equitable immunisation coverage is the most effective way to prevent transmission in post-elimination contexts such as Australia [17,26,30,31,32]. We demonstrate that national and international measles immunisation targets were achieved, with higher coverage among Aboriginal children. Nonetheless, areas of under-immunisation were identified [4,7,17]. Contrary to common assumptions, increased rurality and the early pandemic period were not associated with lower immunisation rates. Receipt of MCV1 was a strong predictor of MCV2 immunisation in our cohort. The 95% MCV2 coverage threshold was achieved at 1582 days, and most children received doses on time. Those who received late doses were largely immunised within six months of their due date. Combining coverage data with timeliness measures provides a clearer picture of true protection in subgroups that may have been obscured in broader geographical analyses.

Factors influencing measles immunisation coverage and timeliness are multifactorial, including health service availability and accessibility, geographic location, cultural safety and responsiveness of services, vaccine confidence, and opportunity for immunisation [31,33]. Identifying and addressing these factors is critical to ensure equitable uptake and to meet immunisation targets through data-driven, community-tailored solutions [26,33]. Even in countries with high overall coverage, heterogeneity in coverage with clustering of under-immunised population groups creates immunity gaps. This leaves children susceptible to measles infection, and subpopulations at risk of sustained transmission [30,32,34,35].

The negative impacts of the COVID-19 pandemic on immunisation rates are well documented, including on health service delivery, immunisation access, and waning vaccine confidence [2,11,17]. Public health social measures in 2020 and 2021 were in place in NSW when children from the 2018 and 2019 birth cohorts were scheduled for measles immunisation. This means that local impacts of the early pandemic could be assessed [36]. Our results do not reflect negative trends and instead challenge prevalent assumptions, with higher coverage and comparable timeliness among children scheduled for their first measles immunisation early in the pandemic, including priority populations, when compared with older cohorts.

National and state policies in Australia may have influenced the improving trend in coverage and timeliness seen in younger birth cohorts, though the extent of this is unknown. The National ‘No Jab, No Pay’ policy was introduced in 2016, and the NSW ‘No Jab, No Play’ policy in 2014, establishing immunisation requirements for family tax benefits and childcare payments, and childcare attendance, respectively [16,37]. Evaluation of these vaccine mandates indicates a positive impact on population immunisation coverage; however, with a negative differential impact on subpopulations (including more socioeconomically disadvantaged groups), this raises ethical considerations [38,39].

The 95% MCV2 coverage threshold was achieved at 1582 days (four years and four months) for the study cohort. We demonstrate that most children received vaccines on time or within a few months of the scheduled due date, with only a small proportion receiving immunisations more than six months late, extending the time until 95% MCV2 coverage is reached. Australia, like many other countries, uses static timepoints to measure immunisation coverage [17]. While national coverage data measures MCV2 at 24 months, the state-based performance measures capture measles coverage at the 12–15 months, 24–27 months and 60–63 months timepoints [17,20]. The 60–63-month timepoint captured when population 95% MCV2 coverage was achieved in our study; however, this timepoint is somewhat after the 24–27 months measure and may have concealed a delay between these timepoints. However, this was not an important finding in this HNELHD cohort of children. The small cohort being immunised more than six months late is likely to benefit most from earlier catch-up, which may subsequently influence achieving earlier 95% MCV2 coverage. Evaluation of immunisation performance tends to focus on coverage rather than timeliness; however, measurements of timeliness provide important insight into the quality of immunisation programmes. Delayed immunisation can impact vaccine protection, which leaves children susceptible during a time of vulnerability to measles complications. This has been associated with not receiving later scheduled immunisations [40,41,42]. Gras et al. propose thresholds for potentially dangerous immunisation delays of over one month for MCV1 and six months for MCV in a French context (with a similar immunisation schedule to Australia) [40]. When categorical timeliness measures such as set timepoints are used, quantification of delay is limited, and insight is lost, including an understanding of potentially dangerous delays [41]. We demonstrate how a model encompassing both categorical and continuous timeliness measures gives greater insight into true population protection [40]. We propose that assessing timeliness by measuring the actual time of immunisation provides the information necessary to inform targeted public health activity. This has potential policy implications insofar as it provides an opportunity for better understanding and to address declining immunisation.

Subpopulation analysis utilising this methodology demonstrated immunisation equity within the HNELHD region. MCV2 coverage amongst Aboriginal children was 98.9% across birth cohorts and geographic regions, which surpassed national coverage rates [17]. On-time immunisation and prompt catch-up within six months of the scheduled date was demonstrated. Initiatives such as employing Aboriginal staff to assess the cultural safety of immunisation services and pre-calling Aboriginal parents to encourage vaccination may have contributed to these results [33,39,43]. An economic gradient in immunisation coverage was not demonstrated, though minimal variations in timeliness may reflect accessibility disparities. Increasing rurality is considered a risk for lower immunisation coverage and health service accessibility [17,31,33]; however, we demonstrated that outer regional areas had equivalent measles immunisation coverage to major cities and only slightly lower on-time immunisation. Whilst this is encouraging for regional service delivery overall, geographic clusters of lower immunisation LGAs support recommendations for more localised data provision [32]. Understanding local barriers to immunisation could inform targeted interventions using the Tailoring Immunisation Programmes to address service delivery, accessibility, and acceptability barriers [30,44]. A potential immunity gap was identified amongst children without Indigenous status recorded, which may reflect inequity in measles immunisation access or incomplete history taking regarding immunisations received overseas and subsequently recorded in the AIR. Many of these children received vaccines overseas, which suggests they may be migrants, refugees, or asylum seekers, also as many reside in a Federal Government Humanitarian Settlement Programme regional location, and with a large international student cohort of child-bearing age attending the University of New England [45,46]. Travel patterns in migrant communities may increase the risk of measles exposure, reinforcing the strategic direction of maintaining high immunisation coverage in this group through targeted interventions [17,44,47].

This study highlights the benefit of detailed, timely, high-quality immunisation data availability for providers, public health authorities, and communities to optimise data-informed immunisation activity [18,19,48,49]. Improving data use to strengthen immunisation outcomes is prioritised in the National Immunisation Strategy for Australia 2025–2030 and the NSW Immunisation Strategy 2024–2028 and aligns with the National Agreement on Closing the Gap [18,19,48]. Collectively, these directions highlight a commitment to data-driven policy that improves programme effectiveness, reduces inequities, and addresses vulnerabilities. We demonstrate how localised data analysis of coverage and timeliness together enabled the identification of subpopulations with under-immunisation, which would otherwise be masked within regional data. This would be further enhanced with near-real-time data for prompt public health activity [33].

Limitations of this study are acknowledged. The data utilised relies on accuracy and completion of documentation, and any gaps may impact the accuracy of coverage estimates. Several factors may contribute to inaccurate data; however, data linkage may offer solutions to improve data completeness [24]. Accurate Aboriginal health statistics depend on reliable Indigenous status reporting supported by culturally safe identification practices, which are essential for estimating immunisation coverage, guiding policy, ensuring culturally appropriate care, and evaluating programmes in line with the National Safety and Quality Health Service Standards [24,43,50]. Using AIR records, which are informed by parent/carer reports to Medicare, aimed to minimise inaccuracy, and we note improving Indigenous data completeness over time; however, there may be residual misclassification of Indigenous status in the study [24]. Coverage estimates may be artificially lowered when children who are no longer eligible for immunisation (e.g., permanently left Australia or died) remain in the AIR without an end date applied to their record. The AIR is considered a near-complete population register (approximately 99% of children are enrolled in Medicare and therefore linked in the AIR by 12 months of age); however, it is possible that a small number of children were not yet registered or were Medicare ineligible and not included in this analysis [25,26]. Delays in updating Medicare details following migration into or out of the region may impact the study population. Ensuring the correct denominators are used requires systematic processes for updating the AIR records to improve accuracy and would allow for better identification of under-immunised populations, informing targeted interventions [24,26]. Measures of ethnicity other than Indigenous status (such as primary language and country of birth) are not captured in the AIR. This limits immunisation data for people from multicultural backgrounds and, while vaccines received overseas can serve as a proxy for migration, this fails to fully capture coverage data for these populations. Vaccines received overseas are ideally entered into the AIR as the date they were received overseas, but records may have been entered differently. Additionally, we acknowledge variations in routine vaccine schedule recommendations globally, which may impact when children receive vaccines, such as MCV1 scheduled prior to 11 months, or MCV2 scheduled later (e.g., Japan, Republic of Korea) [51]. Doses given prior to 11 months (for travel or overseas scheduling) are recommended to be followed up routinely by MCV1 and MCV2 [4,7]. Our study cohort included only children born in the first five months of 2019, limiting comparisons with birth cohorts comprising a full year.

## 5. Conclusions

The high and timely coverage with measles immunisation in HNELHD children, spanning cultural, social, and geographic subgroups, challenges universal assumptions about the negative early impacts of the COVID-19 pandemic. They also provide encouraging reassurance regarding local efforts to close the immunisation gap for Aboriginal children. However, relatively small subpopulation immunity gaps were identified, which may be masked within sub-national level data.

Current state-based performance measures based on static timepoints capture when the 95% MCV2 coverage threshold is achieved; however, they might be too late to inform meaningful earlier intervention to improve measles coverage. We demonstrate the additional value of combining categorical and continuous timeliness data and propose this as a useful adjunct to static coverage data to provide a better understanding of protection against measles.

Improving immunisation data availability, accessibility, and timeliness, including through appropriate data linkage, is consistent with National and State strategic priorities. This offers the potential to better inform targeted public health activity and thus improve measles immunisation coverage and timeliness, ultimately better protecting communities.

## Figures and Tables

**Table 1:** Data variables included in the study and their data sources.

Variable	Definition
Date of birth	Standard from AIR
Indigenous status	Standard from AIR and informed by Medicare records as reported by the child’s parent or guardian. Children were described as identifying as Indigenous, non-Indigenous or Indigenous status not recorded. The classification of Indigenous includes children who identify as Aboriginal, Torres Strait Islander, and both Aboriginal and Torres Strait Islander *.
Postcode	Standard from AIR.
Date of valid MCV1	The Australian Immunisation Handbook recommends MCV1 at 12 months of age; however, doses given from 11 months and one day old are valid doses as they are sufficiently immunogenic and would not require repeat immunisation. Doses received prior to 11 months of age (for example, for infants travelling overseas) were not considered valid, as two subsequent routine doses are recommended [7].
Date of valid MCV2	MCV2 is recommended to be given at 18 months of age according to the Australian Immunisation Handbook [7]. MCV2 was considered valid if it was received more than 30 days after a valid MCV1 dose was given, irrespective of the timing of MCV1. The minimum dosing interval between MCV1 and MCV2 is 30 days in Australia.
On-time immunisation for MCV1 **	Vaccines were considered on time when they were given within 30 days of being due. For MCV1, a vaccine given on time could be given from 11 months and one day old until 12 months and 30 days old.
On-time immunisation for MCV2 **	On time valid MCV2 was determined based on when MCV1 was received. For children who received MCV1 on time, MCV2 was on time if given within 30 days of 18 months of age. MCV2 doses received before 18 months old, but after the minimum interval of 30 days after MCV1, were also considered on time. For children who received their MCV1 late, MCV2 was considered on time if given 30 days after the first dose.
Local government area (LGA)	Derived from postcode. There are 22 LGAs within HNELHD, described by name.
SEIFA # decile	Derived from postcode.
ARIA ^ area	Derived from postcode.

* Within NSW Health, the term Aboriginal is generally used in preference to “Aboriginal and Torres Strait Islander” in recognition that Aboriginal people are the original inhabitants of NSW. Therefore, the term Aboriginal is respectfully used to include all Aboriginal and Torres Strait Islander peoples in the analysis. ** Overdue doses for immunisations were determined using the Australian Immunisation Register National due and overdue rules for immunisation [20]. # SEIFA = Socio-Economic Index For Australia. ^ ARIA = Accessibility/Remoteness Index of Australia.

**Table 2:** Demographic details of children included in the study.

Demographic	Total Cohort
Birth cohort	
2015	12,454 (23.3%)
2016	12,412 (23.3%)
2017	11,931 (22.4%)
2018	11,645 (21.8%)
2019 *	4948 (9.3%)
Indigenous status	
Aboriginal	7864 (14.7%)
Non-Indigenous	45,189 (84.6%)
Not recorded	337 (0.6%)
Local Government Area	
Armidale	1586 (3.0%)
Cessnock	4099 (7.7%)
Dungog	525 (1.0%)
Glen Innes Severn	402 (0.8%)
Gunnedah	897 (1.7%)
Gwydir	248 (0.5%)
Inverell	1003 (1.9%)
Lake Macquarie	11,976 (22.4%)
Liverpool Plains	391 (0.7%)
Maitland	5995 (11.2%)
Mid-Coast	4238 (7.9%)
Moree Plains	798 (1.5%)
Muswellbrook	1021(1.9%)
Narrabri	802 (1.5%)
Newcastle	8662 (16.2%)
Port Stephens	3793 (7.1%)
Singleton	1507 (2.8%)
Tamworth	3976 (7.5%)
Tenterfield	250 (0.5%)
Upper Hunter	768 (1.4%)
Uralla	308 (0.6%)
Walcha	145 (0.3%)
ARIA	
Major cities	30,732 (57.6%)
Inner regional	18,124 (33.9%)
Outer regional	4534 (8.5%)
SEIFA decile	
1	4284 (8.0%)
2	7977 (14.9%)
3	2586 (4.8%)
4	11,666 (21.9%)
5	7492 (14.0%)
6	14,327 (26.8%)
7	1761 (3.3%)
8	2584 (4.8%)
9	713 (1.3%)
Total	53,390

* The 2019 birth cohort includes children born until 1 June 2019.

## Data Availability

The datasets presented in this article are not readily available because they are provided to the HNELHD Public Health Unit for service review and quality assurance purposes. Requests to access the dataset should be directed to the AIR.

## Abbreviations

The following abbreviations are used in this manuscript:

AIR	Australian Immunisation Register
ARIA	Accessibility/Remoteness Index of Australia
HNELHD	Hunter New England Local Health District
LGA	Local government area
MCV	Measles-containing vaccine
MCV1	Measles-containing vaccine dose one
MCV2	Measles-containing vaccine dose two
MMR	Measles, mumps, and rubella vaccine
MMRV	Measles, mumps, rubella and varicella vaccine
SEIFA	Socio-Economic Index For Australia
WHO	World Health Organization

## Appendix A

**Table A1:** Median age and interquartile range, Kruskal–Wallis test results, and post hoc analysis results of MCV1 and MCV2 by (a) birth cohort and (b) Indigenous status for HNELHD children in the study cohort.

(a) Birth Cohort										
MCV Dose	Birth Cohort	Median Age (IQR) (Days)	Kruskal–Wallis T	Post Hoc Comparisons Indicating Younger Age at Immunisation *						
				2015	2016		2017	2018		2019
MCV1	2015	375 (369–390)	χ^2^(4) = 62.2, *p* < 0.01	-						
	2016	375 (369–390)			-					
	2017	375 (369–391)					-			
	2018	374 (368–389)						-		
	2019	373 (369–386)		*p* < 0.01	*p* < 0.01		*p* < 0.01	*p* < 0.01		-
MCV2	2015	560 (551–583)	χ^2^(4) = 17.2, *p* < 0.01	-						
	2016	560 (551–584)			-					
	2017	560 (551–583)					-			
	2018	559 (551–583)						-		
	2019	560 (551–583)			*p* < 0.01		*p* < 0.01	*p* < 0.01		-
**(b) Indigenous Status**										
**MCV Dose**	**Indigenous Status**	**Median Age (IQR) (Days)**	**Kruskal–Wallis T**	**Post Hoc Comparisons Indicating Younger Age at Immunisation ***						
				Aboriginal		Non-Indigenous			Not recorded	
MCV1	Aboriginal	376 (369–397)	χ^2^(2) = 148.9, *p* < 0.01	-					*p* < 0.01	
	Non-Indigenous	374 (369–388)		*p* < 0.01		-			*p* < 0.01	
	Not Recorded	401 (372–566)							-	
MCV2	Aboriginal	562 (551–590)	χ^2^(2) = 146.3, *p* < 0.01	-					*p* < 0.01	
	Non-Indigenous	559 (551–582)		*p* < 0.01		-			*p* < 0.01	
	Not Recorded	605 (558–1726)							-	

* *p*-values for statistically significant comparisons are shown. Non-statistically significant comparisons have been left blank.
